# Racial differences in kidney cancer histology and outcome: A nationwide study from the UroCCR Cohort

**DOI:** 10.1002/cncr.70120

**Published:** 2025-10-08

**Authors:** Xiaofan Lu, Jean‐Christophe Bernhard, Gaëlle Margue, François Audenet, Nicolas Doumerc, Pierre Merlin, Morgan Roupret, Thibaut Waeckel, Cécile Champy, Louis Surlemont, Jonathan Olivier, Nicolas Branger, Bastien Parier, Victor Gaillard, Franck Bruyere, Alexis Fontenil, Constance Michel, Louis Vignot, Jean‐Luc Descotes, Guillaume Loison, Julien Guillotreau, Fayek Taha, Frédéric Panthier, Stéphane De Vergie, Idir Ouzaid, Romain Boissier, Jean‐Jacques Patard, Pierre Gimel, Igor Duquesne, Khalil Chalhoub, Pierre Bigot, Gabriel G. Malouf

**Affiliations:** ^1^ Department of Cancer and Functional Genomics Institute of Genetics and Molecular and Cellular Biology CNRS/INSERM/UNISTRA Illkirch France; ^2^ Department of Urology CHU Bordeaux Bordeaux France; ^3^ Department of Urology Hôpital Européen Georges Pompidou Paris France; ^4^ Department of Urology CHU Rangueil Toulouse France; ^5^ Department of Urology Hôpital Lyon Sud ‐ Hospices Civils de Lyon Oullins‐Pierre‐Bénite France; ^6^ Department of Urology GRC 5 Predictive Onco‐Urology Sorbonne University Hôpital Pitié‐Salpêtrière AP–HP Paris France; ^7^ Department of Urology CHU Caen Caen France; ^8^ Department of Urology CHU Henri‐Mondor AP‐HP Creteil France; ^9^ Department of Urology CHU Rouen Rouen France; ^10^ Department of Urology CHRU Lille Lille France; ^11^ Department of Urology Institut Paoli Calmettes Marseille France; ^12^ Department of Urology Hôpital Bicêtre AP‐HP Le Kremlin‐Bicêtre Paris France; ^13^ Department of Urology CHRU Strasbourg Strasbourg France; ^14^ Department of Urology CHRU Tours Tours France; ^15^ Department of Urology CHU Nîmes Nîmes France; ^16^ Department of Urology Hospital Paris Saint‐Joseph Paris France; ^17^ Department of Urology CHU Nice Nice France; ^18^ Department of Urology CHU Grenoble La Tronche France; ^19^ Department of Urology Clinique La Croix du Sud Quint‐Fonsegrives France; ^20^ Department of Urology Clinique Pasteur Toulouse France; ^21^ Department of Urology CHU Reims Reims France; ^22^ Department of Urology Hôpital Tenon AP‐HP Paris France; ^23^ Department of Urology CHU Nantes Nantes France; ^24^ Department of Urology Hôpital Bichat Claude‐Bernard AP‐HP Paris France; ^25^ Department of Urologic Surgery and Renal Transplantation CHU Conception AP‐HM Aix Marseille Université Marseille France; ^26^ Department of Urology CH Mont‐de‐Marsan Mont‐de‐Marsan France; ^27^ Department of Urology Médipôle Saint‐Roch ‐ Clinique Privé Cabestany Cabestany France; ^28^ Department of Urology Hôpital Cochin AP‐HP Paris France; ^29^ Department of Urology CH Kourou Kourou France; ^30^ Department of Urology CHU Angers Angers France; ^31^ Department of Medical Oncology Strasbourg University Institut de Cancérologie de Strasbourg Strasbourg France

**Keywords:** differential expression, molecularly defined histologies, racial differences, renal cell carcinoma, universal health care system

## Abstract

**Introduction:**

Racial differences in renal cell carcinoma (RCC) have been well described in U.S. cohorts, particularly for common histologic subtypes. However, such differences remain underexplored in European populations, especially for rare RCC subtypes and within universal health care settings. We aimed to characterize racial differences in RCC histology, renal comorbidities, and clinical outcomes in France, and to investigate potential molecular underpinnings.

**Methods:**

A total of 9404 patients were analyzed from the UroCCR national registry (1987–2024), including 338 Black and 9066 non‐Black individuals with nephrectomy‐confirmed diagnoses. Clinical features, histologic subtypes, and outcomes were compared between groups. Transcriptomic differences between Black and White individuals were assessed using publicly available single‐nucleus and bulk RNA‐sequencing data from nonneoplastic renal tissue.

**Results:**

Clear‐cell RCC was less common in Black patients (47.6% vs. 68.8%), whereas papillary, translocation, and fumarate hydratase–deficient RCC subtypes, as well as angiomyolipoma, were more prevalent (all odds ratios ≥ 2). Black patients were diagnosed younger, with smaller, earlier‐stage tumors, and underwent partial nephrectomy more frequently. No difference in disease‐specific survival was observed; however, distant recurrence‐free survival was longer in Black patients (hazard ratio, 0.54; *p* = .019), likely reflecting earlier diagnosis. Race was not associated with survival after adjusting for clinical factors. Transcriptomic profiling revealed upregulation of adipogenic and angiogenic programs in White individuals and enrichment of hemoglobin‐ and chemokine‐related pathways in Black individuals. Limitations include retrospective design and race classification.

**Conclusion:**

Distinct racial patterns in RCC histology and renal biology may inform subtype susceptibility. These findings support ancestry‐informed strategies in RCC diagnosis and research.

## Abstract 2

Black patients in France exhibit a higher prevalence of non–clear cell renal cell carcinoma subtypes and are diagnosed at younger ages with earlier‐stage tumors. These patterns, along with distinct transcriptomic profiles in nonneoplastic renal tissue, support ancestry‐informed approaches to renal cell carcinoma diagnosis and research.

## Plain Language Summary

This study examines racial differences in kidney cancer in France using data from more than 9000 patients who underwent surgery. It finds that Black patients are more likely to have rare and aggressive non–clear cell kidney cancers, but are diagnosed at younger ages and earlier stages, leading to better short‐term outcomes. Importantly, survival differences between racial groups disappeared after accounting for clinical factors. The study also explores gene activity in normal kidney tissues and finds biological differences that may help explain cancer subtype susceptibility. These findings support the need for ancestry‐informed approaches in kidney cancer diagnosis and research.

## INTRODUCTION

Renal cell carcinoma (RCC) is a biologically and clinically heterogeneous disease with notable variation in incidence, prognosis, and treatment response across racial groups.^1^ Globally, RCC incidence has risen, with clear racial differences.^2^ For instance, clear cell RCC (ccRCC) is more common in White patients, whereas papillary RCC (PRCC) and molecularly defined subtypes, such as fumarate hydratase (FH)–deficient RCC and translocation RCC (TRCC), are more frequently seen in Black individuals.^3^, ^4^, ^5^ These subtype patterns may partly explain observed disparities in disease aggressiveness and outcomes.^3^, ^4^, ^5^

Most data on racial differences come from U.S.‐based registries such as SEER and the National Cancer Database, which have identified contributing risk factors including male sex, chronic kidney disease (CKD), hypertension, and obesity.^6^, ^7^, ^8^ However, their generalizability to European populations—particularly within universal health care systems—is unclear.^9^, ^10^ France, with its ethnically diverse population and equal‐access health care, provides a unique setting to study RCC differences. In 2023, ∼17,000 kidney cancer cases were diagnosed nationally, approximately 25% of which were metastatic, yet large‐scale data disaggregated by race remain lacking.^11^, ^12^

Emerging genomic evidence suggests ancestry‐related tumor features in RCC. In ccRCC, Black patients show lower rates of von Hippel‐Lindau (VHL) mutations and reduced hypoxia‐inducible factor (HIF) signaling.^13^ A genome‐wide association study in African Americans identified a protective variant at 11q13.3 (rs7105934) that reduces ccRCC risk by altering HIF‐2α binding.^14^ Yet, the transcriptomic landscape of nonneoplastic renal tissue across racial groups remains poorly characterized, despite its potential role in shaping tumor susceptibility.

To address these gaps, we analyzed racial differences in RCC subtype distribution, kidney comorbidities, and survival in a large, demographically annotated, nephrectomy‐based cohort from France (UroCCR‐191 project). We further integrated transcriptomic data from nonneoplastic renal epithelium to explore underlying biological variation. Together, these findings provide an epidemiologic and molecular framework for ancestry‐informed kidney cancer research and care.

## MATERIALS AND METHODS

### Study population

This study used data from the UroCCR database (NCT03293563), a French national registry of kidney cancer. The database is institutional review board–approved and holds CNIL authorization (DR‐2013‐206), with ethical oversight from the Committee for the Protection of Persons South‐West and Overseas III (decision number DC 2012/108) and CCTIRS. It is supported by the French National Cancer Institute, the French High Authority of Health, GIS‐IBiSA, and the French National Research Agency.

The primary study population included patients who underwent either partial or radical nephrectomy across 30 participating tertiary centers between December 1987 and May 2024. Eligibility required a confirmed histological diagnosis of kidney cancer or a related renal condition. All participants received written information and gave informed consent, in accordance with the Declaration of Helsinki. For secondary analyses on advanced disease, we included additional patients with biopsy‐confirmed histologic diagnoses without nephrectomy, as recorded in UroCCR.

### Racial classification

Race was determined by clinicians or research staff using medical chart documentation, consistent with standard UroCCR procedures. This classification reflected contextual background assessed during clinical intake, rather than self‐report.

### Inclusion and exclusion criteria

Among 10,149 cases with pathology records, we excluded those lacking a confirmed diagnosis or detectable tumor (*n* = 56), unknown race (*n* = 17), undefined RCC subtype (*n* = 21), rare or unspecified renal conditions with <30 cases (*n* = 496), mixed ccRCC and non‐ccRCC diagnoses (*n* = 126), or multiple non‐ccRCC subtypes (*n* = 29). The final cohort included 9404 patients: 338 Black and 9066 non‐Black individuals.

### Demographic and clinical variables

Variables included age, sex, body mass index, comorbidities, histologic subtype, symptoms at diagnosis, surgery type, TNM stage, Fuhrman grade, metastasis sites, diagnosis date, and survival outcomes.

### Transcriptomic data analysis

To examine racial differences in nontumor renal transcriptomes, we used version 1.5 of the Healthy and Injured Human Kidney Atlas, analyzing ∼304,000 cells via single‐nucleus RNA‐sequencing (snRNA‐seq) data from version 1.5 of the Healthy and Injured Human Kidney Atlas, which includes approximately 304,000 cells.^15^ We generated pseudo‐bulk gene expression by aggregating renal epithelial transcripts. Because of the limited availability of histologically normal kidney tissue from Black donors and to reduce confounding by tissue type, we retained only samples from injured kidneys (20 Black and 30 White individuals). Differential expression was performed using DESeq2 (v1.38.0) and MOVICS (v0.99.17);^16^, ^17^ pathway enrichment used clusterProfiler (v4.6.0) with MSigDB gene sets.^18^ For validation, we analyzed bulk RNA‐seq data from The Cancer Genome Atlas (TCGA) KIRC, KIRP, and KICH projects, focusing on 118 nontumor samples (eight Black, 110 White). Batch effects were corrected using sva (v3.46.0), followed by differential expression with DESeq2.

### Statistical analysis

Analyses were conducted in R (v4.2.2). Continuous variables were summarized as medians with interquartile ranges (IQRs); group comparisons used Mann–Whitney U and Fisher exact tests. Missing data were assumed completely at random and excluded casewise. Racial differences in disease prevalence were assessed via 2×2 contingency tables and odds ratios (OR) using Fisher exact test. A Haldane‐Anscombe correction (adding 0.5) was used for cells with zero counts.

Survival outcomes were analyzed with Kaplan–Meier curves and log‐rank tests (survival, v3.4.0). Cox models estimated hazard ratios (HRs) with 95% CIs. The primary endpoint was disease‐specific survival (DSS), defined as time from diagnosis to kidney cancer–related death. Patients alive or dying from other causes were censored at last follow‐up. The secondary endpoint was distant recurrence‐free survival (DRFS), assessed in nonmetastatic patients (M0), and defined as time from diagnosis to first distant metastasis. Patients who died without recurrence were censored at death.

The *p* values were adjusted for multiple testing using the Benjamini‐Hochberg false discovery rate (FDR), except in multivariable Cox models that assess the joint effects of covariates. For all unadjusted analyses, a 2‐sided *p* < .05 was considered statistically significant.

## RESULTS

### Comprehensive cohort description

**FIGURE 1:**
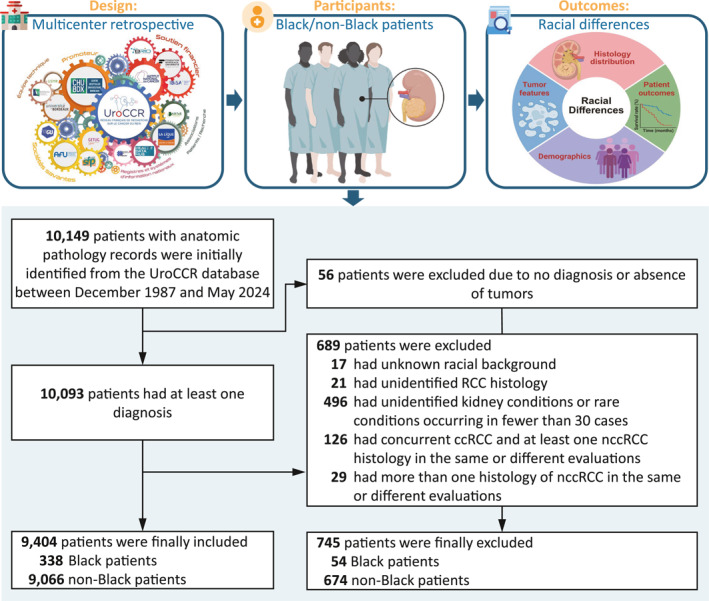
Flow diagram of inclusions and exclusions using the UroCCR database.

**FIGURE 2:**
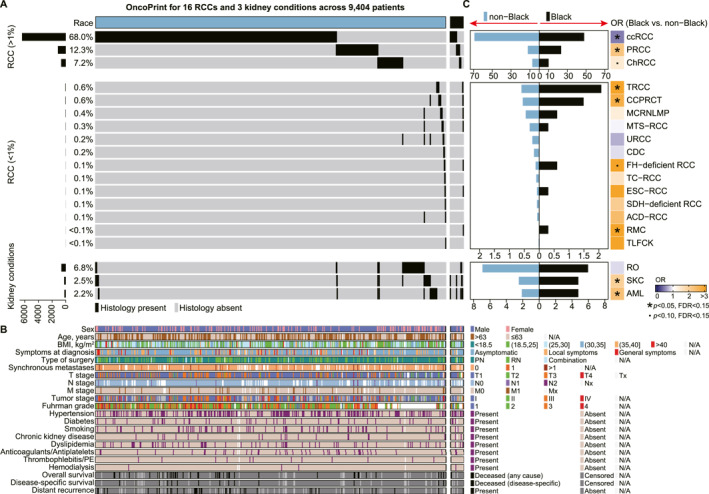
Racial differences in histological distribution. (A) OncoPrint illustrating the incidence and distribution of 16 renal cell carcinoma (RCC) types and three kidney conditions across 9404 patients. (B) Heatmap showing baseline characteristics of the study population. (C) Bar plot displaying the histological incidence in non‐Black versus Black patients, with odds ratios (ORs) calculated for each histology. Statistical significance is denoted, derived from a Fisher exact test.

Of 10,149 patients enrolled in the UroCCR registry (1987–2024), 745 were excluded, resulting in a final analytic cohort of 9404 patients, all of whom underwent either partial or radical nephrectomy (Figure 1). Among these, 8483 had RCC across 16 histologic subtypes (Figure 2A). CcRCC was most common (68%), followed by PRCC (12.3%) and chromophobe RCC (ChRCC) (7.2%). Less common subtypes included TRCC and clear cell papillary renal cell tumor (CCPRCT) (0.7% and 0.6%, respectively). Other rare histologies (each <0.5% of cases) included FH‐deficient RCC, collecting duct carcinoma (CDC), mucinous tubular and spindle cell carcinoma, unclassified RCC, eosinophilic solid and cystic RCC, renal medullary carcinoma (RMC), tubulocystic RCC, acquired cystic disease‐associated RCC, thyroid‐like follicular carcinoma of the kidney, succinate dehydrogenase–deficient RCC, and multilocular cystic renal neoplasm of low malignant potential. Additionally, 921 patients were diagnosed with kidney‐relevant conditions, including renal oncocytoma (6.8%), simple kidney cysts (SKC, 2.5%), and angiomyolipoma (AML, 2.2%). Some patients presented with both RCC and these conditions.

The cohort comprised 9066 non‐Black and 338 Black patients (Figure 2B, Table S1), with a male predominance (67.1%) and median age of 63 years (IQR: 54‐71). Most were overweight (38.2%) or obese (25.5%), consistent with known RCC risk factors.^19^ Common comorbidities included hypertension (46.8%), diabetes (16.7%), smoking (19.3%), and CKD (7.2%); dyslipidemia (20.3%), anticoagulant use (12.9%), and hemodialysis (1.4%) were less frequent. Compared with the general French population (Table S2), obesity, hypertension, diabetes, anticoagulant use, and hemodialysis were more prevalent. In contrast, daily smoking was less common, and the prevalence of CKD and dyslipidemia was comparable.

At diagnosis, 69.0% were asymptomatic; 24.2% had local and 6.8% systemic symptoms. Partial nephrectomy was performed in 62.6%, radical nephrectomy in 35.2%. Most tumors were T1a (45.4%) or T3a (24%), with N0 (94.9%) and M0 (92.2%) predominating. Stage I was most frequent (60.9%), followed by III (22.4%), IV (9.9%), and II (6.8%). Fuhrman grades showed 44.3% with moderate differentiation (grade 2) and 49.9% with high‐grade tumors (grades 3‐4). Among 8476 patients with follow‐up (median: 28.5 months), 1063 deaths occurred, including 563 disease‐specific. One‐, 2‐, and 3‐year DSS was 98.2%, 96.0%, and 94.0%, respectively.

### Racial differences in disease incidence

Marked differences in histologic subtype distribution were observed between Black and non‐Black patients (Figure 2C). CcRCC was significantly less common in Black patients (47.6% vs. 68.8%; OR, 0.41; *p* < .001; FDR < 0.001), whereas PRCC was more common (23.1% vs. 11.8%; OR, 2.23; *p* < .001; FDR < 0.001). ChRCC showed marginally increased incidence (9.8% vs. 7.1%; OR, 1.42; *p* = .068; FDR = 0.143). Several rare subtypes were also enriched in Black patients, including TRCC (2.1% vs. 0.6%; OR, 3.60; *p* = .005; FDR = 0.021), CCPRCT (1.5% vs. 0.5%; OR, 2.76; *p* = .044; FDR = 0.119), RMC (0.3% vs. 0; OR, 80.59; *p* = .001; FDR = 0.008), and FH‐deficient RCC (0.6% vs. 0.1%; OR, 5.39; *p* = .067; FDR = 0.143).

Overall, nccRCC comprised 44.7% of diagnoses in Black patients vs. 23.9% in non‐Black (*p* < .001), highlighting a pronounced racial difference (Figure 2C). This pattern was also seen among excluded 728 cases: Black patients were overrepresented (7.4% vs. 3.6%; OR, 2.14; *p* < .001), reflecting the disproportionate exclusion of complex histologies more common in this group.

Benign renal conditions were also more frequent in Black patients (Figure 2C), including SKC (4.7% vs. 2.4%; OR, 1.98; *p* = .019; FDR = 0.062) and AML (4.7% vs. 2.1%; OR, 2.33; *p* = .003; FDR = 0.016).

### Racial differences in baseline characteristics

Significant demographic and clinical differences emerged between Black and non‐Black patients (Table 1). Black patients were diagnosed at a younger median age (*p* < .001, FDR < 0.001), suggesting earlier disease onset. This difference persisted across key RCC subtypes (i.e., ccRCC, PRCC, and ChRCC) and benign kidney conditions (all, FDR < 0.05), with similar trends in rare subtypes including TRCC and FH‐deficient RCC (both, *p* < .1, FDR < 0.15), suggesting earlier onset is not solely from subtype distribution (Figure S1). Obesity was more common among Black patients (body mass index > 30 kg/m^2^: 30.6% vs. 25.4%; *p* = .043, FDR = 0.060).

**TABLE 1:** Racial differences in baseline characteristics.

	Non‐Black, *n* = 9066	Black, *n* = 338	*p* ^a^	FDR	Missing, %^b^
Age at diagnosis (years), median [IQR]	63 [54, 71]	58.00 [48, 66]	<.001	<0.001	1.2
Sex, *n* (%)			.448	0.462	0
Male	6094 (67.2)	220 (65.1)			
Female	2972 (32.8)	118 (34.9)			
Body mass index (kg/m^2^), *n* (%)			.025	0.038	3.9
Below 18.5, underweight	142 (1.6)	6 (1.9)			
18.5–24.9, normal	3018 (34.7)	105 (32.5)			
25.0–29.9, overweight	3336 (38.3)	113 (35.0)			
30.0–34.9, obesity I	1484 (17.0)	73 (22.6)			
35.0–39.9, obesity II	508 (5.8)	24 (7.4)			
Above 40, obesity III	217 (2.5)	2 (0.6)			
Obesity (body mass index > 30 kg/m^2^)			.043	0.060	3.9
Present	2213 (25.4)	99 (30.6)			
Absent	6500 (74.6)	225 (69.4)			
Hypertension, *n* (%)			.072	0.096	0.5
Present	4208 (46.6)	172 (51.8)			
Absent	4815 (53.4)	160 (48.2)			
Diabetes, *n* (%)			.004	0.009	0.5
Present	1489 (16.5)	75 (22.6)			
Absent	7534 (83.5)	257 (77.4)			
Smoking, *n* (%)			.006	0.011	0.5
Present	1760 (19.5)	44 (13.3)			
Absent	7263 (80.5)	288 (86.7)			
Chronic kidney disease, *n* (%)			<.001	<0.001	0.5
Present	627 (6.9)	45 (13.6)			
Absent	7184 (79.6)	275 (82.8)			
Dyslipidemia, *n* (%)			.174	0.198	0.5
Present	1839 (20.4)	57 (17.2)			
Absent	7184 (79.6)	275 (82.8)			
Anticoagulants/antiplatelets, *n* (%)			.004	0.008	0.5
Present	1186 (13.1)	25 (7.5)			
Absent	7837 (86.9)	307 (92.5)			
Thrombophlebitis/pulmonary embolism, *n* (%)			.346	0.369	0.5
Present	350 (3.9)	9 (2.7)			
Absent	8673 (96.1)	323 (97.3)			
Hemodialysis, *n* (%)			.004	0.008	0.5
Present	116 (1.3)	11 (3.3)			
Absent	8907 (98.7)	321 (96.7)			
Symptoms at diagnosis, *n* (%)			.282	0.311	2.2
Asymptomatic	6115 (68.9)	233 (71.9)			
Local/general symptoms	2755 (31.1)	91 (28.1)			
Type of surgery, %			.002	0.006	1.4
Partial nephrectomy	5576 (62.3)	230 (70.6)			
Radical nephrectomy	3173 (35.5)	95 (29.1)			
Combination	197 (2.2)	1 (0.3)			
Synchronous metastases, *n* (%)			.007	0.012	20
0	6777 (93.5)	262 (97.8)			
≥1	475 (6.5)	6 (2.2)			
T, *n* (%)			<.001	<0.001	2.9^c^
T1 + T2	6327 (71.8)	271 (85.2)			
T3 + T4	2487 (28.2)	47 (14.8)			
N, *n* (%)			.147	0.181	21.9^c^
N0	6735 (94.8)	235 (97.1)			
N1 + N2	369 (5.2)	7 (2.9)			
M, *n* (%)			.001	0.004	7.6^c^
M0	7705 (92.0)	302 (97.1)			
M1	672 (8.0)	9 (2.9)			
Tumor stage, *n* (%)			<.001	<0.001	22.2
I + II	4751 (67.2)	205 (85.1)			
III + IV	2323 (32.8)	36 (14.9)			
Fuhrman grade, *n* (%)			.003	0.007	19
1 + 2	3665 (49.8)	151 (59.4)			
3 + 4	3697 (50.2)	103 (40.6)			
Overall survival, *n* (%)			.023	0.038	9.9
Censored	7149 (87.3)	264 (92.0)			
Deceased (any cause)	1040 (12.7)	23 (8.0)			
Disease‐specific survival, *n* (%)			.113	0.145	9.9
Censored	7638 (93.3)	275 (95.8)			
Deceased (disease‐specific)	551 (6.7)	12 (4.2)			
Distant recurrence, *n* (%)			.001	0.004	16.8^d^
Present	905 (12.0)	15 (5.4)			
Absent	6642 (88.0)	264 (94.6)			

*Note*: Continuous variables were summarized using medians and interquartile ranges (IQR). Categorical variables were presented as proportions, with any missing data excluded from the respective calculations.

^a^ *p* values were calculated using the Mann–Whitney *U* test for continuous data and Fisher exact test for categorical data.

^b^ Missing rates were presented for each variable where data was unavailable.

^c^ Missing rates for TNM stages are represented by Tx, Nx, and Mx, respectively.

^d^ Distant recurrence was calculated for patients without confirmed metastasis at presentation (M0 or Mx) and patients who had M1 tumors were classified as missing data.

Black patients had a higher burden of comorbidities. Diabetes (22.6% vs. 16.5%; *p* = .004, FDR = 0.009), CKD (13.6% vs. 6.9%; *p* < .001, FDR < 0.001), and hemodialysis use (3.3% vs. 1.3%; *p* = .004, FDR = 0.008) were more prevalent. Hypertension was more frequent (51.8% vs. 46.6%) but not statistically significant (*p* = .072, FDR = 0.096). Smoking (13.3% vs. 19.5%; *p* = .006, FDR = 0.011) and anticoagulant use (7.5% vs. 13.1%; *p* = .004, FDR = 0.008) were lower among Black patients; the latter may influence perioperative risk profiles.

Black patients more often received partial nephrectomy (70.6% vs. 62.3%; *p* = .002, FDR = 0.006), which is supported by a higher prevalence of smaller tumors (T1–T2: 85.2% vs. 71.8%; *p* < .001, FDR < 0.001), earlier‐stage disease (Stage I–II: 85.1% vs. 67.2%; *p* < .001, FDR < 0.001), and more favorable tumor grade (Fuhrman 1–2: 59.4% vs. 49.8%; *p* = .003, FDR = 0.007). They were also less likely to present with metastases (M0: 97.1% vs. 92.0%; *p* = .001, FDR = 0.004).

These favorable baseline characteristics aligned with better outcomes: lower distant recurrence (5.4% vs. 12.0%; *p* = .001, FDR = 0.004), and overall mortality (8.0% vs. 12.7%; *p* = .023, FDR = 0.038). Disease‐specific mortality was lower in Black patients, but not statistically significant (4.2% vs. 6.7%; *p* = .113, FDR = 0.145).

### Racial differences in survival

**FIGURE 3:**
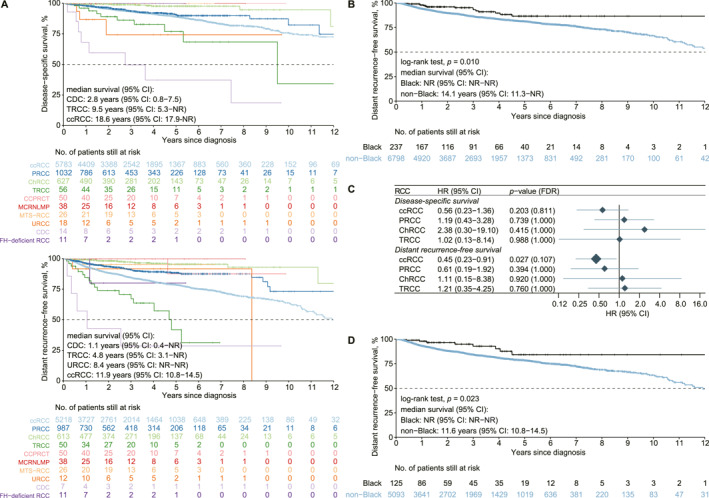
Racial differences in patient outcomes. (A) Kaplan–Meier curves in upper panel showing disease‐specific survival rates for major RCC histologies (*n* > 10), whereas lower panel showing distant recurrence‐free survival rates. Median survival was calculated for each histology. (B) Kaplan–Meier curves comparing distant recurrence‐free survival rates between Black and non‐Black patients across all RCC histologies. (C) Forest plot illustrating hazard ratios (HR, with 95% CIs) for several RCC histologies of interest through univariate Cox proportional hazards regression models; *p* values were adjusted to control the false discovery rate (FDR). (D) Kaplan–Meier curves comparing distant recurrence‐free survival rates between Black and non‐Black patients specifically for ccRCC histology. ccRCC indicates clear cell renal cell carcinoma; RCC, renal cell carcinoma.

Survival outcomes varied by RCC subtype (Figure 3A). Among subtypes with >10 cases, CDC had the shortest median DSS at 2.8 years, followed by TRCC (9.5 years) and ccRCC (18.6 years). Median DSS was not reached for other non‐ccRCCs. In terms of DRFS, CDC again showed the poorest with median 1.1 years, followed by TRCC (4.8 years), unclassified RCC (8.4 years), and ccRCC (11.9 years). Overall, patients with non‐ccRCC subtypes demonstrated more favorable outcomes compared to those with ccRCC (Figure S2A–B).

Stratified by race, DSS did not differ significantly (*p* = .429). but DRFS was significantly longer for Black patients (*p* = .010) (Figure 3B). Among major subtypes (ccRCC, PRCC, ChRCC, TRCC), DSS was similar across races (all, *p* > .2, FDR > 0.8) (Figure 3C), but Black patients with ccRCC had longer DRFS (HR, 0.45; 95% CI, 0.23–0.91; *p* = .027; FDR = 0.107) (Figure 3C). Median DRFS was 11.6 years for non‐Black patients but not reached in Black patients (*p* = .023) (Figure 3D), indicating this subtype largely accounted for overall DRFS difference.

Univariate analysis confirmed no association between race and DSS (*p* = .328, FDR = 0.424; Table S3), but a lower risk of distant recurrence in Black patients (HR, 0.54; 95% CI, 0.33–0.91; *p* = .019; FDR = 0.032) (Table 2). Factors linked to poor DSS included older age, smoking, anticoagulant use, thromboembolic events, radical nephrectomy, advanced stage, and high grade (Table S3).

**TABLE 2:** Hazard ratios using univariate and multivariate Cox regression models for predictors of distant recurrence‐free survival.

Variable^a^	Univariate Cox regression			Multivariate Cox regression	
	Unadjusted HR (95% CI)	*p*	FDR	Adjusted HR (95% CI)	*p*
Race		.019	0.032		.894
Non‐Black (ref.)	1.00			1.00	
Black	0.54 (0.33–0.91)			0.96 (0.5–1.80)	
Age (years)		<.001	<0.001		.007
≤63 (ref.)	1.00			1.00	
>63	1.49 (1.30–1.71)			1.24 (1.06–1.45)	
Sex		.001	0.003		.003
Female (ref.)	1.00			1.00	
Male	1.28 (1.10–1.50)			1.30 (1.09–1.54)	
Obesity (BMI > 30 kg/m^2^)		.792	0.839		–
Absent (ref.)	1.00			–	
Present	1.02 (0.87–1.19)			–	
Type of nephrectomy^b^		<.001	<0.001		<.001
Partial (ref.)	1.00			1.00	
Radical	6.14 (5.26–7.16)			2.99 (2.47–3.63)	
T		<.001	<0.001		<.001
T1 + T2 (ref.)	1.00			1.00	
T3 + T4	6.50 (5.62–7.52)			2.38 (1.98–2.87)	
N		<.001	<0.001		<.001
N0 (ref.)	1.00			1.00	
N1 + N2	7.02 (5.65–8.72)			2.82 (2.23–3.56)	
Tumor stage		<.001	<0.001		–^d^
I + II (ref.)	1.00			–	
III + IV	6.35 (5.40–7.46)			–	
Fuhrman grade		<.001	<0.001		<.001
1 + 2 (ref.)	1.00			1.00	
3 + 4	4.75 (4.01–5.63)			2.40 (1.97–2.92)	
Histology^c^		<.001	<0.001		.062
ccRCC (ref.)	1.00			1.00	
nccRCC	0.46 (0.38–0.55)			0.81 (0.65–1.01)	
Hypertension		.155	0.215		–
Absent (ref.)	1.00			–	
Present	1.10 (0.96–1.26)			–	
Diabetes		.475	0.535		–
Absent (ref.)	1.00			–	
Present	1.07 (0.89–1.27)			–	
Smoking		.005	0.009		.335
Absent (ref.)	1.00			1.00	
Present	1.25 (1.07–1.47)			1.09 (0.91–1.30)	
Chronic kidney disease		.888	0.888		–
Absent (ref.)	1.00			–	
Present	1.02 (0.79–1.32)			–	
Dyslipidemia		.265	0.341		–
Absent (ref.)	1.00			–	
Present	1.10 (0.93–1.29)			–	
Anticoagulants/antiplatelets		<.001	<0.001		.863
Absent (ref.)	1.00			1.00	
Present	1.46 (1.22–1.74)			1.02 (0.84–1.24)	
Thrombophlebitis/pulmonary embolism		.449	0.535		–
Absent (ref.)	1.00			–	
Present	1.13 (0.83–1.54)			–	
Hemodialysis		.091	0.136		–
Absent (ref.)	1.00			–	
Present	0.50 (0.22–1.12)			–	

Abbreviations: BMI, body mass index; FDR, false discovery rate; HR, hazard rate.

^a^ Patients with any missing data relevant to the variable being analyzed were excluded.

^b^ Patients undergoing combination surgery types were excluded.

^c^ Patients with only kidney conditions (i.e., renal oncocytoma, simple kidney cysts, or angiomyolipoma) were excluded.

^d^ Tumor stage was excluded from the multivariate analysis to prevent multicollinearity.

Obesity was associated with better DSS (HR, 0.78; 95% CI, 0.63–0.96; *p* = .021; FDR = 0.036), consistent with the “obesity paradox.”^20^, ^21^ This benefit was seen in ccRCC (*p* = .006), but not non‐ccRCC (*p* = .938) (Figure S3A‐B).

Similarly, older age, male sex, smoking, anticoagulant or antiplatelet use, radical nephrectomy, and more advanced tumor stage and grade were all associated with poorer DRFS (all, FDR < 0.01; Table 2). Multivariate analysis confirmed that higher T stage, N stage, Fuhrman grade, older age, male sex, and radical nephrectomy were all significantly associated with worse DRFS (all, *p* < .01, FDR < 0.01). Race was not significant after adjustment (*p* = .894), suggesting racial differences in recurrence risk were mediated by tumor characteristics rather than race per se (*p* = .894) (Table 2).

To assess whether racial differences in survival extend to more advanced disease, we additionally analyzed 806 patients with biopsy‐ or nephrectomy‐confirmed stage IV RCC (11 Black, 795 White). This extended cohort had a significantly higher prevalence of metastatic disease compared to the main nephrectomy cohort (16.5% vs. 9.9%, *p* < .0001). Non–clear cell histologies remained more frequent in Black patients (3.8% vs. 0.99%, *p* = .042), but race was not associated with DSS (*p* = .207, FDR = 0.357) (Table S4). These results reinforce our primary conclusion that race is not an independent predictor of survival in RCC and that observed differences are more likely explained by tumor characteristics.

### Inherent racial transcriptomic differences in nonneoplastic injured renal tissues

**FIGURE 4:**
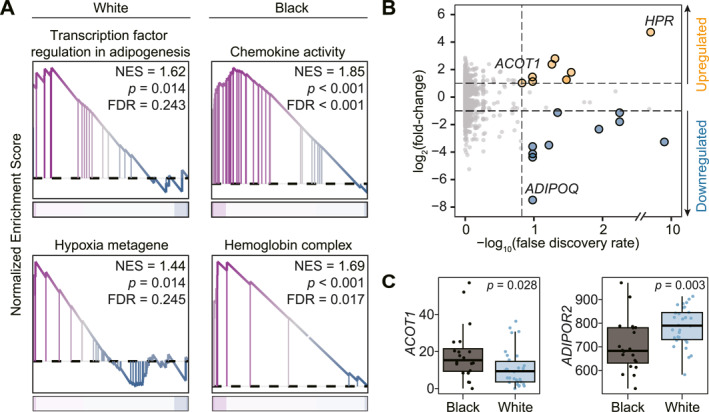
Inherent transcriptomic differences in nonneoplastic injured renal tissues from Black vs. White individuals. (A) Gene set enrichment analysis of pseudo‐bulked renal epithelial cells from a single‐nucleus RNA‐sequencing dataset comparing 20 Black and 30 White individuals. Gene sets were considered significantly enriched at *p* < .05 and FDR < 0.25. The left panel shows gene sets upregulated in White individuals; the right panel shows those upregulated in Black individuals. (B) Volcano plot of differentially expressed genes from bulk RNA‐sequencing of histologically normal kidney tissue adjacent to RCC in TCGA (Black: *n* = 8; White: *n* = 110). Genes with |log_2_ fold‐change| > 1, *p* < .001, and FDR < 0.15 are color‐coded and considered significantly differentially expressed. (C) Boxplots of normalized count expression for *ACOT1* (left) and *ADIPOR2* (right) in the snRNA‐seq dataset, comparing Black and White individuals. Statistical significance was assessed using the two‐sample Mann–Whitney *U* test. FDR indicates false discovery rate; RCC, renal cell carcinoma; snRNA‐seq, single‐nucleus RNA‐sequencing.

To investigate transcriptomic differences in nonneoplastic renal tissue between racial groups, we analyzed a snRNA‐seq dataset comprising renal epithelial cells from 20 Black and 30 White individuals with injured kidneys. Pseudo‐bulk analysis revealed distinct expression patterns. White individuals showed increased adipogenesis and hypoxia pathway activity; Black individuals had enrichment in chemokine signaling and hemoglobin complex formation (Figure 4A). To validate these patterns, we examined bulk RNA‐seq data from 118 adjacent normal kidney samples in TCGA (Black: *n* = 8; White: *n* = 110). Despite the heterogeneity of bulk tissue, differential expression analysis confirmed several genes with race‐specific patterns (fold‐change > 2, *p* < .001; FDR < 0.15) (Figure 4B). Among the most strongly upregulated genes in Black samples was *HPR*, which encodes a hemoglobin‐binding protein. *ACOT1*, an acyl‐CoA thioesterase, was also consistently upregulated in Black individuals in both datasets (Figure 4C). In White individuals, *ADIPOQ*, a classic adipocyte marker, and its receptor *ADIPOR2* were more highly expressed in White individuals (Figure 4C).

## DISCUSSION

To our knowledge, this is the largest demographically annotated, nephrectomy‐based RCC cohort to examine racial differences in RCC incidence and histologic distribution in a French population. Although previous studies focused mainly on major subtypes,^22^, ^23^ we included rare and recently defined RCC entities. We confirmed that PRCC and ChRCC are more frequently diagnosed in Black patients, whereas ccRCC is less common. We also observed enrichment of CCPRCT, a morphologically ambiguous subtype sharing features of ccRCC and PRCC, among Black individuals.

Importantly, we also examined molecularly defined RCC subtypes often underreported in registry‐based cohorts due to rarity or diagnostic complexity. TRCC, FH‐deficient RCC, and RMC were more common in Black patients. Our finding of increased TRCC incidence is consistent with previous U.S. cohorts.^4^ Although the underlying mechanism remains unclear, potential explanations include ancestry‐related biological differences, as suggested by recent genomic studies comparing African and European RCC populations.^3^

In FH‐deficient RCC, both genetic and environmental factors likely contribute. A recent meta‐analysis showed that African ancestry is associated with higher rates of pathogenic FH germline variants.^24^ These tumors display fumarate‐driven metabolic dysregulation, global DNA hypermethylation, and pseudohypoxia.^25^ Additionally, concurrent *NF2* truncating mutations, which are linked to synchronous metastases and bone involvement, appear more prevalent in Black patients.^25^ Enrichment of COSMIC signature SBS22, associated with aristolochic acid exposure, further suggests a gene–environment interaction.^25^

RMC exhibits one of the most pronounced ancestry‐specific patterns, occurring almost exclusively in individuals with sickle cell trait or disease—conditions more prevalent in people of African descent.^26^ This highly rare and aggressive tumor is characterized by early onset and *SMARCB1* loss, likely driven by chronic hypoxic stress in the renal medulla from sickling‐induced ischemia.^27^

We also reported a higher likelihood of Black patients harboring conditions like SKC and AML, the latter driven by mutations in *TSC1*/*TSC2*.^28^ SKC has been linked to early kidney injury and comorbidities such as albuminuria, hypertension, and hyperfiltration,^29^ which is consistent with the greater burden of hypertension and CKD in Black patients.

Clinically, Black patients presented with younger age, smaller tumors, and earlier stage disease, resulting in more partial nephrectomies and favorable unadjusted outcomes. These findings mirror U.S. studies reporting earlier‐stage RCC in Black patients.^30^, ^31^, ^32^, ^33^, ^34^, ^35^ Notably, age differences persisted within specific subtypes—ccRCC, PRCC, and ChRCC—suggesting that early onset is not merely driven by enrichment of well‐known early‐onset RCC subtypes (e.g., TRCC, FH‐RCC, RMC). Although rates of symptomatic presentation were similar across races, the age gap may reflect genetic predisposition, tumor biology, or earlier incidental detection due to more frequent comorbidities (e.g., hypertension, diabetes, CKD) in Black patients. However, these possibilities require further investigation.

In multivariable models adjusting for clinical factors, race was not independently associated with DSS or DRFS. The improved DRFS in Black patients in unadjusted analyses appears to be mediated by more favorable baseline characteristics, rather than reflecting inherent biological differences by race. These findings contrast with several U.S.‐based studies that reported worse survival among Black patients, often in the context of systemic disparities in access to care, diagnostic delays, or treatment inequities.^36^, ^37^, ^38^, ^39^ Although we did not directly assess health care access, the lack of racial disparities in survival may reflect more equitable treatment within France’s universal health care system. This is consistent with equal‐access U.S. cohorts where survival differences were also attenuated.^40^, ^41^

To further explore biological underpinnings, we analyzed snRNA‐seq data from injured nonneoplastic kidneys. White individuals showed upregulation of adipogenesis and hypoxia pathways—hallmarks of ccRCC initiation.^42^ Notably, *ADIPOQ* and its receptor *ADIPOR2* were more highly expressed, pointing to a lipid‐enriched epithelial environment permissive to ccRCC. In contrast, Black individuals exhibited upregulation of chemokine signaling and hemoglobin complex genes. Elevated chemokine activity suggests a more inflamed epithelial state, as seen in immune‐infiltrated RCCs such as RMC and CDC.^27^, ^43^ Increased hemoglobin gene expression may reflect tubular hemoglobin reabsorption or subclinical hemolysis related to sickle cell trait, a known risk factor for RMC.^26^ This profile might also indicate a tissue environment shaped by chronic epithelial stress or recurrent injury, aligning with epidemiological evidence linking acute kidney injury to subsequent development of PRCC.^44^ Additionally, consistent upregulation of *ACOT1*—a thioesterase that hydrolyzes long‐chain acyl‐CoAs and limits substrate availability for lipid biosynthesis—may further suppress adipogenic signaling, opposing the lipid‐rich phenotype characteristic of ccRCC.

Several limitations warrant mention. First, our nephrectomy‐based design excluded patients not eligible for surgery, potentially underrepresenting advanced‐stage cases. Second, recruitment from tertiary centers may introduce referral and consent bias. Third, the rarity of certain subtypes and the limited number of Black patients, especially in rare histologies, restricted statistical power for some analyses. Fourth, racial classification grouped diverse non‐Black populations together and likely included individuals of sub‐Saharan African or Caribbean descent in the Black group, and White, Asian, and other ancestries in the non‐Black group, potentially masking intragroup heterogeneity. Finally, our transcriptomic analyses were constrained by sample availability and tissue context. The TCGA validation cohort included only eight Black patients and relied on histologically normal tissue adjacent to tumors, which may be influenced by the tumor microenvironment. Similarly, our snRNA‐seq analysis used injured kidney tissue, which may not accurately reflect steady‐state epithelial biology. Future studies leveraging healthy, spatially resolved, and cell‐type–specific transcriptomic data across diverse ancestries are needed to fully elucidate race‐associated renal biology.

## CONCLUSIONS

This study reveals distinct histologic and clinical features of RCC among Black patients in France, including a higher prevalence of non–clear cell subtypes. Transcriptomic profiling of nonneoplastic renal tissue identified differential activation of adipogenic, hypoxia, inflammatory, and hemoglobin‐related pathways that may contribute to these differences. Although race was not a prognostic factor after adjusting for clinical variables, our findings support the value of ancestry‐informed cancer research and highlight the need for improved diagnostic strategies to detect rare RCC subtypes.

## AUTHOR CONTRIBUTIONS

All authors contributed to the study design, hypothesis generation, data interpretation, and critical review of the manuscript. Xiaofan Lu developed the computational code and conducted the statistical analyses. Xiaofan Lu, Jean‐Christophe Bernhard, and Gaëlle Margue prepared the tables and figures and drafted the initial version of the manuscript. All authors contributed to subsequent revisions, critically reviewed the methods and results, and approved the final version. Xiaofan Lu, Francois Audenet, Nicolas Doumerc, Pierre Merlin, and Morgan Roupret accessed and verified the data. Pierre Bigot and Gabriel G. Malouf supervised and managed the project. All authors had access to all reported data and held final responsibility for the decision to submit for publication.

## CONFLICT OF INTEREST STATEMENT

Consulting: MSD, EISAI, and IPSEN. Research funding: MSD‐AVENIR.

## Supporting information

Supplementary Material

## Data Availability

The UroCCR data supporting the findings of this study are not publicly available due to institutional and ethical regulations. However, deidentified individual participant data may be made available upon reasonable request to the corresponding author (G.M.) for research purposes, subject to approval. Data access will require completion of a formal data request process, including a data‐sharing agreement to ensure compliance with privacy and ethical considerations. Requests should be directed to g.malouf@icans.eu.
