# Supplementary material for: Racial differences in kidney cancer histology and outcome: A nationwide study from the UroCCR Cohort
Source: Cancer. 2025 Oct 8;131(20):e70120. doi: 10.1002/cncr.70120 (PMC12506607; doi:10.1002/cncr.70120)
Supplement: Supplementary file 1 — Supplementary Material [file CNCR-131-e70120-s001.pdf]

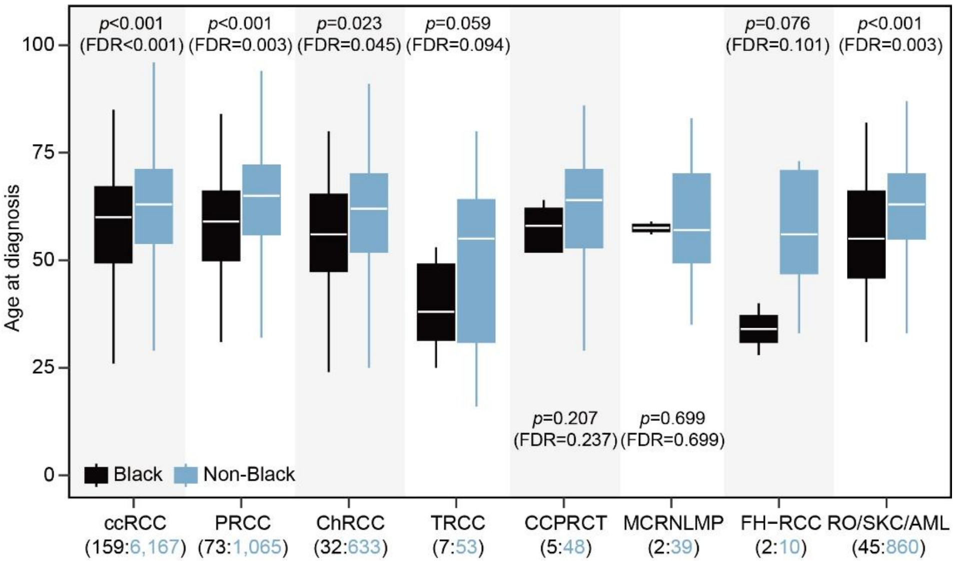

Figure S1. Age at diagnosis by RCC histologies and race.

Boxplots show age distribution at diagnosis among Black and non-Black patients across RCC subtypes and benign kidney conditions. Subtypes included in the analysis had ≥2 cases from each racial group. The number of Black and non-Black patients for each subtype is indicated below the x-axis. *P*-values were calculated using the Mann-Whitney U test, with corresponding false discovery rates (FDR) adjusted.

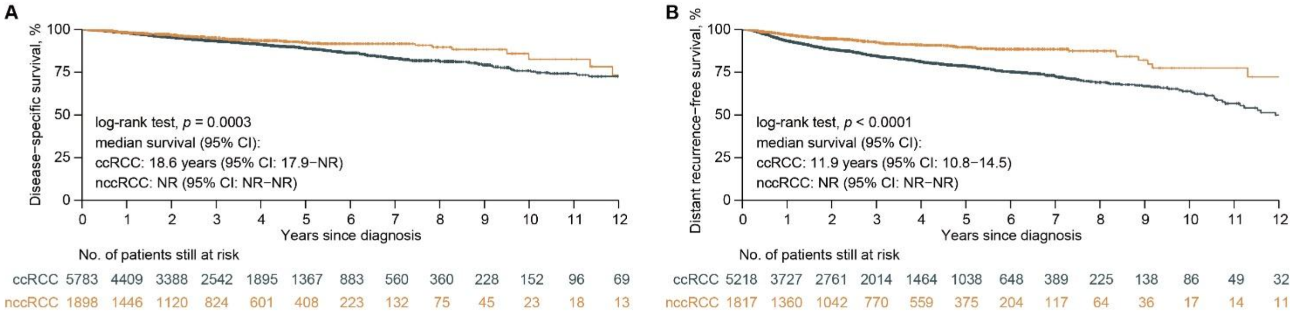

Figure S2. Survival differences between clear cell and non-clear cell histologies

A, Kaplan-Meier curves comparing disease-specific survival rates for patients with clear cell RCC (ccRCC) versus those with non-clear cell RCC (nccRCC). **B**, Same as A but for distant recurrence-free survival.

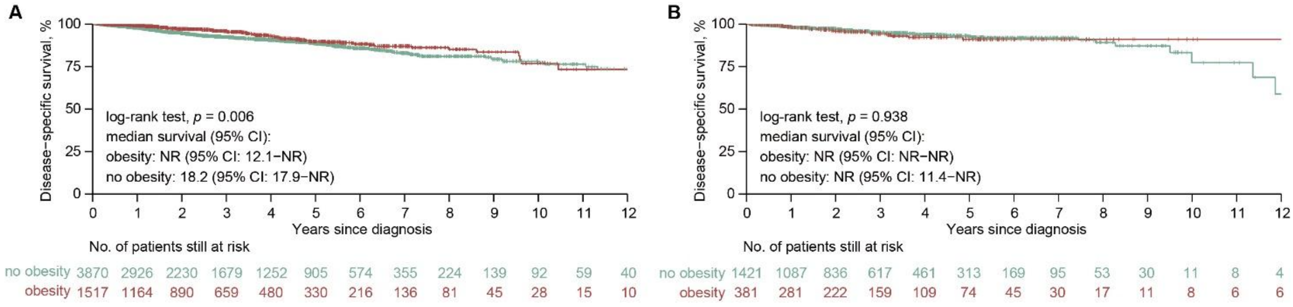

Figure S3. Paradoxical effect of obesity on disease-specific survival between clear cell and non-clear cell histologies

A, Kaplan-Meier curves comparing disease-specific survival rates for clear cell RCC patients with obesity versus those without obesity. **B**, Same as A but for non-clear cell RCC patients.

Table S1. Baseline characteristics

	Overall, n = 9404	non-Black, n = 9066	Black, n = 338	Missing, %[a]
Age at diagnosis (years), median [IQR]	63 [54, 71]	63 [54, 71]	58.00 [48, 66]	1.2
Sex, n (%)				0
Male	6314 (67.1)	6094 (67.2)	220 (65.1)	
Female	3090 (32.9)	2972 (32.8)	118 (34.9)	
BMI (kg/m^2), n (%)				3.9
Below 18.5, underweight	148 (1.6)	142 (1.6)	6 (1.9)	
18.5-24.9, normal	3123 (34.6)	3018 (34.7)	105 (32.5)	
25.0-29.9, overweight	3449 (38.2)	3336 (38.3)	113 (35.0)	
30.0-34.9, obesity I	1557 (17.2)	1484 (17.0)	73 (22.6)	
35.0-39.9, obesity II	532 (5.9)	508 (5.8)	24 (7.4)	
Above 40, obesity III	219 (2.4)	217 (2.5)	2 (0.6)	
Hypertension, n (%)				0.5
Present	4380 (46.8)	4208 (46.6)	172 (51.8)	
Absent	4975 (53.2)	4815 (53.4)	160 (48.2)	
Diabetes, n (%)				0.5
Present	1564 (16.7)	1489 (16.5)	75 (22.6)	
Absent	7791 (83.3)	7534 (83.5)	257 (77.4)	
Smoking, n (%)				0.5
Present	1804 (19.3)	1760 (19.5)	44 (13.3)	
Absent	7551 (80.7)	7263 (80.5)	288 (86.7)	
Chronic kidney disease, n (%)				0.5
Present	672 (7.2)	627 (6.9)	45 (13.6)	
Absent	7459 (79.7)	7184 (79.6)	275 (82.8)	
Dyslipidemia, n (%)				0.5
Present	1896 (20.3)	1839 (20.4)	57 (17.2)	
Absent	7459 (79.7)	7184 (79.6)	275 (82.8)	
Anticoagulants/Antiplatelets, n (%)				0.5
Present	1211 (12.9)	1186 (13.1)	25 (7.5)	
Absent	8144 (87.1)	7837 (86.9)	307 (92.5)	
Thrombophlebitis/Pulmonary embolism, n (%)				0.5
Present	359 (3.8)	350 (3.9)	9 (2.7)	
Absent	8996 (96.2)	8673 (96.1)	323 (97.3)	
Hemodialysis, n (%)				0.5
Present	127 (1.4)	116 (1.3)	11 (3.3)	
Absent	9228 (98.6)	8907 (98.7)	321 (96.7)	
Symptoms at diagnosis, n (%)				2.2
Asymptomatic	6348 (69.0)	6115 (68.9)	233 (71.9)	
Local symptoms	2222 (24.2)	2146 (24.2)	76 (23.5)	
General symptoms	624 (6.8)	609 (6.9)	15 (4.6)	
Type of surgery, %				1.4
Partial nephrectomy	5806 (62.6)	5576 (62.3)	230 (70.6)	
Radical nephrectomy	3268 (35.2)	3173 (35.5)	95 (29.1)	

Combination	198 (2.1)	197 (2.2)	1 (0.3)	
Synchronous metastases, n (%)				20
0	7039 (93.6)	6777 (93.5)	262 (97.8)	
1	324 (4.3)	320 (4.4)	4 (1.5)	
>1	157 (2.1)	155 (2.1)	2 (0.7)	
T, n (%)				2.9[b]
T1	27 (0.3)	27 (0.3)	0 (0.0)	
T1a	4145 (45.4)	3961 (44.9)	184 (57.9)	
T1b	1733 (19.0)	1676 (19.0)	57 (17.9)	
T2	5 (0.1)	4 (0.0)	1 (0.3)	
T2a	492 (5.4)	473 (5.4)	19 (6.0)	
T2b	196 (2.1)	186 (2.1)	10 (3.1)	
T3	6 (0.1)	6 (0.1)	0 (0.0)	
T3a	2190 (24.0)	2146 (24.3)	44 (13.8)	
T3b	206 (2.3)	206 (2.3)	0 (0.0)	
T3c	45 (0.5)	45 (0.5)	0 (0.0)	
T4	87 (1.0)	84 (1.0)	3 (0.9)	
N, n (%)				21.9[b]
N0	6970 (94.9)	6735 (94.8)	235 (97.1)	
N1	268 (3.6)	267 (3.8)	1 (0.4)	
N2	108 (1.5)	102 (1.4)	6 (2.5)	
M, n (%)				7.6[b]
M0	8007 (92.2)	7705 (92.0)	302 (97.1)	
M1	681 (7.8)	672 (8.0)	9 (2.9)	
Tumor stage, n (%)				22.2
I	4456 (60.9)	4272 (60.4)	184 (76.3)	
II	500 (6.8)	479 (6.8)	21 (8.7)	
III	1638 (22.4)	1612 (22.8)	26 (10.8)	
IV	721 (9.9)	711 (10.1)	10 (4.1)	
Fuhrman grade, n (%)				19
1	441 (5.8)	414 (5.6)	27 (10.6)	
2	3375 (44.3)	3251 (44.2)	124 (48.8)	
3	2620 (34.4)	2536 (34.4)	84 (33.1)	
4	1180 (15.5)	1161 (15.8)	19 (7.5)	
Overall survival, n (%)				9.9
Censored	7413 (87.5)	7149 (87.3)	264 (92.0)	
Deceased (any cause)	1063 (12.5)	1040 (12.7)	23 (8.0)	
Disease-specific survival, n (%)				9.9
Censored	7913 (93.4)	7638 (93.3)	275 (95.8)	
Deceased (disease-specific)	563 (6.6)	551 (6.7)	12 (4.2)	
Distant recurrence, n (%)				16.8[c]
Present	920 (11.8)	905 (12.0)	15 (5.4)	
Absent	6906 (88.2)	6642 (88.0)	264 (94.6)	

Continuous variables were summarized using medians and interquartile ranges (IQR). Categorical variables were presented as proportions, with any missing data excluded from the respective calculations.

[a]Missing rates were presented for each variable where data was unavailable.
[b]Missing rates for TNM stages are represented by Tx, Nx, and Mx, respectively.
[c]Distant recurrence was calculated for patients without confirmed metastasis at presentation (M0 or Mx) and patients had M1 tumors were classified as missing data.

Table S2. Comparison of comorbidity prevalence between the UroCCR-191 cohort and the general French population

Comorbidity	UroCCR-191	General French Population	Source & Note
Obesity	25.5%	17.0%	Estimated from 9,598 participants in France, 2020 [1]
Hypertension	46.8%	17.4%	CONSTANCES cohort, 11,112 participants, up to 2019 [2]
Diabetes	16.7%	8.6%	National adult estimate, ~46 million adults in France, 2021 [3]
Smoking	19.3%	25.3%	Estimated from 24,514 participants, daily smoking, France, 2021 [4]
Chronic kidney disease	7.2%	6.26%	CONSTANCES cohort, 11,429 participants, all CKD stages [5]
Dyslipidemia	20.3%	23.3%	Esteban study, 2,011 participants, 2014–2016 [6]
Anticoagulant use	12.9%	4.5%	Based on over 3 million users in France in 2013 (~66 million total population) [7]
Hemodialysis	1.4%	0.07%	Based on 47,324 patients receiving dialysis in 2019 (France population ~66.6 million) [8]

Table S3. Hazard ratios using univariate Cox regression model for predictors of disease-specific survival

Variable[a]	Unadjusted HR (95% CI)	*p*	FDR
Race		0.328	0.424
non-Black (ref.)	1.00		
Black	0.74 (0.41-1.35)		
Age at diagnosis (years)		<0.001	<0.001
≤63 (ref.)	1.00		
>63	1.45 (1.22-1.72)		
Sex		0.777	0.869
Female (ref.)	1.00		
Male	0.97 (0.81-1.17)		
Obesity (BMI > 30 kg/m^2)		0.021	0.036
Absent (ref.)	1.00		
Present	0.78 (0.63-0.96)		
Type of nephrectomy[b]		<0.001	<0.001
Partial (ref.)	1.00		
Radical	11.27 (8.66-14.67)		
T		<0.001	<0.001
T1 + T2 (ref.)	1.00		
T3 + T4	8.59 (6.93-10.64)		

N		<0.001	<0.001
N0 (ref.)	1.00		
N1 + N2	7.37 (5.98-9.10)		
M		<0.001	<0.001
M0 (ref.)	1.00		
M1	8.70 (7.31-10.36)		
Tumor stage		<0.001	<0.001
I + II	1.00		
III + IV	13.28 (10.04-17.56)		
Fuhrman grade		<0.001	<0.001
1 + 2 (ref.)	1.00		
3 + 4	9.13 (6.78-12.29)		
Histology[c]		<0.001	<0.001
ccRCC (ref.)	1.00		
nccRCC	0.68 (0.54-0.85)		
Hypertension		0.125	0.183
Absent (ref.)	1.00		
Present	1.14 (0.96-1.36)		
Diabetes		0.934	0.981
Absent (ref.)	1.00		
Present	0.99 (0.78-1.25)		
Smoking		<0.001	<0.001
Absent (ref.)	1.00		
Present	1.60 (1.33-1.93)		
Chronic kidney disease		0.453	0.538
Absent (ref.)	1.00		
Present	1.13 (0.82-1.57)		
Dyslipidemia		0.981	0.981
Absent (ref.)	1.00		
Present	1.00 (0.81-1.24)		
Anticoagulants/Antiplatelets		0.015	0.028
Absent (ref.)	1.00		
Present	1.33 (1.06-1.67)		
Thrombophlebitis/Pulmonary embolism		0.032	0.051
Absent (ref.)	1.00		
Present	1.46 (1.03-2.07)		
Hemodialysis		0.334	0.424
Absent (ref.)	1.00		
Present	0.62 (0.23-1.65)		
[a]Patients with any missing data relevant to the variable being analyzed were excluded.			
[b]Patients undergoing combination surgery types were excluded.			
[c]Patients with only kidney conditions (i.e., RO, SKC or AML) were excluded.			

Table S4. Hazard ratios using univariate Cox regression model for predictors of disease-specific survival in stage IV diseases

Variable[a]	Unadjusted HR (95% CI)	*p*	FDR
Race		0.207	0.357
non-Black (ref.)	1.00		
Black	1.68 (0.75-3.78)		
Age at diagnosis (years)		0.147	0.315
≤63 (ref.)	1.00		
>63	1.19 (0.94-1.49)		
Sex		0.720	0.831
Female (ref.)	1.00		
Male	0.96 (0.75-1.22)		
Obesity (BMI > 30 kg/m^2)		0.398	0.597
Absent (ref.)	1.00		
Present	0.89 (0.67-1.17)		
T		<0.001	<0.001
T1 + T2 (ref.)	1.00		
T3 + T4	1.94 (1.44-2.61)		
N		<0.001	<0.001
N0 (ref.)	1.00		
N1 + N2	2.04 (1.57-2.64)		
Fuhrman grade		<0.001	0.002
1 + 2 (ref.)	1.00		
3 + 4	2.17 (1.42-3.33)		
Histology[b]		0.006	0.017
ccRCC (ref.)	1.00		
nccRCC	1.55 (1.14-2.12)		
Hypertension		0.214	0.357
Absent (ref.)	1.00		
Present	1.16 (0.92-1.45)		
Diabetes		0.455	0.620
Absent (ref.)	1.00		
Present	0.88 (0.64-1.22)		
Smoking		<0.001	0.002
Absent (ref.)	1.00		
Present	1.53 (1.20-1.94)		
Chronic kidney disease		0.546	0.682
Absent (ref.)	1.00		
Present	1.17 (0.70-1.98)		
Dyslipidemia		0.089	0.222
Absent (ref.)	1.00		
Present	0.77 (0.58-1.04)		
Anticoagulants/Antiplatelets		0.839	0.899
Absent (ref.)	1.00		
Present	0.96 (0.68-1.37)		

Thrombophlebitis/Pulmonary embolism		0.957	0.957
Absent (ref.)	1.00		
Present	0.99 (0.57-1.69)		
[a]Patients with any missing data relevant to the variable being analyzed were excluded.			
[b]Patients with only kidney conditions (i.e., RO, SKC or AML) were excluded.			

References

1. Fontbonne A, Currie A, Tounian P, et al. Prevalence of Overweight and Obesity in France: The 2020 Obepi-Roche Study by the "Ligue Contre l'Obésité". J Clin Med. 2023;12.

2. Cheddani L, Lelong H, Goldberg M, Zins M, Blacher J, Kab S. Prediction of incidence of hypertension in France and associated factors: results from the CONSTANCES cohort. Journal of Hypertension. 2025;43: 976-985.

3. Federation ID. IDF Diabetes Atlas. 11th ed: International Diabetes Federation.

4. Pasquereau A, Andler R, Guignard R, et al. Prévalence nationale et régionale du tabagisme en France en 2021 parmi les 18-75 ans, d'après le Baromètre de Santé publique France. Bull Épidémiol Hebd. 2022;26: 470-480.

5. Blacher J, Kab S, Cheddani L, et al. Prevalence of chronic kidney disease in France - The constances cohort. BMC Nephrol. 2025;26: 312.

6. Blacher J, Gabet A, Vallée A, et al. Prevalence and management of hypercholesterolemia in France, the Esteban observational study. Medicine (Baltimore). 2020;99: e23445.

7. Jourdi G, Mansour A, Vayne C, et al. Anticoagulation therapy in France: state-of-the-art in 2020. Annals of Blood. 2020;5.

8. Canaud B, Couchoud C. Global Dialysis Perspective: France. Kidney360. 2022;3: 168-175.
